# Cardiopulmonary resuscitation performed by trained providers and shorter time to emergency medical team arrival increased patients’ survival rates in Istra County, Croatia: a retrospective study

**DOI:** 10.3325/cmj.2019.60.325

**Published:** 2019-08

**Authors:** Katerina Bakran, Andrej Šribar, Monika Šerić, Gordana Antić-Šego, Marija Ana Božić, Aleksandra Prijić, Taša Lacković, Jasminka Peršec

**Affiliations:** 1 Clinical Department for Anesthesiology, Reanimatology and Intensive Care Medicine, University Hospital Dubrava, Zagreb, Croatia; 2 Institute of Emergency Medicine of Istra County, Pula, Croatia; 3 Department of Physical Medicine and Rehabilitation, General Hospital Pula, Pula, Croatia; 4 Department of Anesthesiology, Reanimatology and Intensive Care, General Hospital Pula, Pula, Croatia; 5 Department of Otorhinolaryngology, General Hospital Pula, Pula, Croatia; 6 School of Dental Medicine, University of Zagreb, Zagreb, Croatia

## Abstract

**Aim:**

To assess the effect of the time for emergency medical services (EMS) arrival on resuscitation outcome in the transition period of the EMS system in Istra County.

**Methods:**

This retrospective study analyzed the data from 1440 patients resuscitated between 2011 and 2017. The effect of demographic data, period of the year, time for EMS arrival, initial cardiopulmonary resuscitation (CPR) provider, initial cardiac rhythm, and airway management method on CPR outcome was assessed with multivariate logistic regression.

**Results:**

Survivors were younger than non-survivors (median of 66 vs 70 years, *P* < 0.001) and had shorter time for EMS arrival (median of 6 vs 8 min, *P* < 0.001). The proportion of non-survivors was significantly higher when initial basic life support (BLS) was performed by bystanders without training (83.8%) or when no CPR was performed before EMS team arrival (87.3%) than when BLS was performed by medical professionals (66.8%) (*P* < 0.001). Sex, airway management, and tourist season had no effect on CPR outcome.

**Conclusion:**

Since the time for arrival and level of CPR provider training showed a significant effect on CPR outcome, further organizational effort should be made to reduce the time for EMS arrival and increase the number of individuals trained in BLS.

Clinicaltrials.gov No. NCT03703531.

The Department of Emergency Medicine of Istra County was founded in the beginning of 2012, during the reorganization of the Croatian emergency care system. It consists of eight local offices distributed geographically in such a way that timely help can be provided to the entire county population of 208 201 (2). Emergency medical services (EMS) teams, made up of a physician, nurse, and driver are directed to the location by a dispatcher who performs initial triage using bystander obtained data.

Several factors contribute to EMS organizational difficulties in Istra County. Due to its location on Istrian peninsula, the County is physically and functionally isolated from mainland Croatia. Another factor is the outdated road network whose modernization has started only recently, but there is still no full-profile highway between Pula, the county capital, and the Učka tunnel connecting Istria and mainland Croatia. Furthermore, most of the population is concentrated in coastal towns, while the inland is not as densely populated. Since Pula County Hospital, the only one in the county, is located at the southwestern tip of the peninsula, there is an asymmetry in hospital distribution, and most of the resuscitated patients during last few years have been transferred to the University Hospital Rijeka (Primorje-Gorski kotar County) due to organizational reasons (possibility of performing percutaneous coronary intervention or cardiac surgical treatment). During tourist season, the county population and traffic intensity triples, especially on the coast, so that local government in some districts funds additional EMS teams (3).

The Department of Emergency Medicine of Istra County started systematically introducing quality control and education of medical professionals at the end of 2012. By the end of 2013, all the EMS providers had passed a two-to-four-day course. During the four-day course, physicians acquired basic knowledge in Advanced Life Support (ALS) (4), International Life Support (ITLS), European Pediatrics Advanced Life Support (EPALS) (5) protocols, and medical documentation writing. The physicians’ theoretical knowledge of protocols and skills was repeatedly re-evaluated, and their practical work was supervised by an emergency medicine specialist.

Out-of-hospital cardiac arrest (OHCA) is one of the most challenging clinical scenarios for emergency medical services because of its dramatic presentation, need for immediate response, and inevitable fatal outcome without adequate and timely treatment (1). If resuscitation is performed immediately after onset, the chance of survival is 3-fold greater, and each minute of delay in CPR initiation reduces patient’s survival chances by 10%-15% (1). Central nervous system is most susceptible to hypoxia/anoxia caused by lack of organ perfusion, and early return of spontaneous circulation is extremely important in achieving satisfactory neurological recovery after OHCA (1). The most prominent mechanism of OHCA is ventricular fibrillation (VF), which originated from untreated ventricular tachycardia (VT), while VF as a primary cause of OHCA is not as common (1).

In order to improve OHCA survival rates, education in bystander CPR and introduction of automated external defibrillators in Istra County was performed gradually. Both of these are important parts of the “chain of survival” (6) concept, leading to better overall outcomes. This study assessed the effect of the time for EMS arrival on resuscitation outcome in the EMS system in Istra County and investigated whether these parameters changed after educational and organizational changes that were introduced in the department in 2012.

## Patients, materials, and methods

This retrospective observational study used data obtained from medical records. The study was approved the Ethics Committee of the Institute of Emergency medicine of Istra County.

Data for 2011 and 2012 were collected from the BIS computer software database (In2 Grupa, Zagreb, Croatia), and each medical resuscitation report was studied individually. At that time there were no accepted guidelines on CPR reporting in the Department. From the 2013 to 2017, the medical documentation was registered in the computer system E-hitna (Rinels d.o.o., Rijeka, Croatia), and all performed resuscitation procedures had to be recorded according to the Utstein registry requirements (8).

Patient’s age and sex, cause of OHCA, initial resuscitation provider, time to EMS arrival, airway management method, initial monitored heart rhythm, and survival data were recorded. Cardiopulmonary resuscitation procedures were performed according to the European Resuscitation Council Advanced Adult Life Support 2010 and 2015 guidelines (4).

### Statistical analysis

Normality of distribution was tested with the Shapiro Wilk test. Numerical values are presented as means with standard deviation or medians with interquartile range, where appropriate. Differences between numerical values were tested with the Mann-Whitney U test or Kruskal-Wallis test, depending on the number of groups. When there were more than two groups, Dwass-Steel-Critchlow-Fingler (DCSF) pairwise comparison was performed. Differences in frequencies between categorical variables were tested with the chi-squared test, and the Fisher exact test was used in 2 × 2 tables. The predictive value of independent variables on survival rates was tested with binomial logistic regression and displayed as log odds and odds ratio. The level of statistical significance was set at *P* < 0.05. Statistical analysis and data visualization were performed with jamovi software package v 0.9.1.4. (*http://www.jamovi.org*).

## Results

Between January 1, 2011 and December 31, 2017, Istra County EMS teams resuscitated 1440 patients (997 or 69.2% men). The mean patients’ age was 67.1 ± 15.8 years. The most CPR procedures were performed in 2015 (271 or 18.8%) and the fewest in 2011 (150 or 10.4%) (Table 1).

**Table 1:** The number of performed procedures and time to emergency medical services arrival from 2011-2017

Year	No. of procedures performed (%)	Time to arrival (minutes)		P*						
		Mean ± standard deviation	Median (interquartile range)	2011	2012	2013	2014	2015	2016	2017
**2011**	150 (10.4)	7.66 ± 5.90	6 (3-10)	x	0.904	<0.001	0.225	0.129	0.272	<0.001
**2012**	179 (12.4)	7.58 ± 6.40	6 (4-10)	0.904	x	<0.001	0.097	0.051	0.081	<0.001
**2013**	166 (11.5)	11.9 ± 8.33	10 (6-16)	<0.001	<0.001	x	<0.001	<0.001	<0.001	0.041
**2014**	222 (15.4)	8.76 ± 8.06	7 (4-13)	0.225	0.097	<0.001	x	0.851	0.841	0.028
**2015**	271 (18.8)	8.68 ± 6.72	8 (4-12)	0.129	0.051	<0.001	0.851	x	0.938	0.021
**2016**	249 (17.3)	9.57 ± 8.76	8 (3-13)	0.272	0.081	<0.001	0.841	0.938	x	0.065
**2017**	203 (14.1)	9.99 ± 7.86	9 (5.5-14)	<0.001	<0.001	0.041	0.028	0.021	0.065	x

*P values at the intersection between rows and column denote difference between row and column years; Kruskal-Wallis test with Dwass-Steel-Critchlow-Fingler pairwise comparison.

At hospital admission there were 246 (17.1%) survivors (defined as alive with electrocardiographic evidence and present pulse at radial or carotid artery) and 1194 (82.9%) non-survivors. Non-survivors were significantly more frequently male (84.3%), older, and waited longer for EMS arrival than survivors (Table 2).

**Table 2:** Differences between survivors and non-survivors in demographics, time to emergency medical services arrival, and the survival rate during and outside of tourist season

	Survivors	Non-survivors	P*
**Sex (female/male), %**	20.1/15.7	79.9/84.3	0.043
**Age, median (range)**	66 (55-76)	70 (59-79)	<0.001
**Time to EMS arrival in minutes, median (range)**^†^	6 (0-10)	8 (5-13)	<0.001
**Tourist season (yes/no), %**	82.4/83.4	17.6/16.6	0.613

*χ^2^ test for categorical variables and Mann-Whitney U test for continuous variables.

†EMS – emergency medical services.

The median time for EMS arrival throughout all 7 years was 8 (IQR 4-13) minutes. In 2013 and 2017, the time for EMS arrival was significantly longer compared with other years (Kruskal-Wallis test, *P* < 0.001 with DCSF pairwise comparison; Table 1).

The proportion of non-survivors was significantly higher when initial basic life support (BLS) was performed by bystanders without BLS training (83.8%) or when none was performed before EMS team arrival (87.3%) than when BLS was performed by medical professionals or bystanders with BLS training (66.8%, χ^2^ test, *P* < 0.001, Table 3).

**Table 3:** Difference in survival rates according to initial CPR provider, airway management method, and initial rhythm at emergency medical services arrival*

	No. (%) of		Total
	non-survivor	survivor	
**Initial CPR provider**			
no BLS performed	592 (87.3)	86 (12.7)	678
bystander CPR	441 (83.8)	85 (16.2)	526
emergency medical services witnessed CPR^‡^	149 (66.8)	74 (33.2)	223
unknown/not documented	12 (92.3)	1 (7.7)	19
**Airway management method**			
endotracheal tube	211 (82.1)	46 (17.9)	257
laryngeal mask	696 (81.6)	157 (18.4)	853
face mask	53 (82.8)	11 (17.2)	64
oropharyngeal tube	59 (86.8)	9 (13.2)	68
nasopharyngeal tube	3 (60)	2 (40)	5
unknown/not documented	172 (89.1)	21 (10.9)	193
**Initial rhythm at emergency medical services arrival**			
asystole^‡^	706 (92.4)	58 (7.6)	764
unknown/not documented^‡^	36 (80)	9 (20)	45
pulseless electrical activity^‡^	224 (83.9)	43 (16.1)	267
VF^‡^	180 (61.2)	114 (38.8)	294
asystole/pulseless electrical activity^‡^	13 (92.9)	1 (7.1)	14
VT/VF^‡^	17 (94.4)	1 (5.6)	18
pacemaker	1 (100)	0 (0)	1
pulseless VT	5 (41.7)	7 (58.3)	12
VT with pulse	1 (33.3)	2 (66.7)	3
bradycardia under 40/min	11 (50)	11 (50)	22
**Total**	**1194 (82.9)**	**246 (17.1)**	**1440**

*CPR – cardiopulmonary resuscitation; BLS – basic life support; VT/VF – ventricular tachycardia/ventricular fibrillation; VT – ventricular tachycardia.

†*P* < 0.001 for EMS witnessed CPR vs other entries, other differences not significant; airway management method (*P* = 0.11 for the entire cohort, no significant differences between entries); initial rhythm after EMS arrival (*P* < 0.001) – χ^2^ test.

‡Significant differences (*P* < 0.05) in survival between entries.

There was no significant difference in survival rates between various airway management methods (endotracheal tube, laryngeal mask, nasopharyngeal and oropharyngeal airway, and simple face mask) (χ^2^ test, *P* = 0.110, Table 3). Patients with VF had significantly higher survival rates (38.8%) than patients with pulseless electrical activity (16.1%) and asystole (7.6%). Survival was even higher in patients with ventricular tachycardia with (66.7%) and without pulse (58.3%, χ^2^ test, *P* < 0.001, Table 3).

Tourist season was defined as the period between June 1 and September 30. During tourist season, time for EMS arrival was significantly longer (median 8 vs 7 min, Mann-Whitney U test, *P* = 0.002). However, there was no significant difference in survival rates between patients who were resuscitated during (17.6%) or outside of (16.6%) tourist season (χ^2^ test, *P* = 0.613). There was a significant trend of increase in survival rates between 2015 and 2017 (2015 – 25.8%, 2016 – 22.5%, 2017 – 19.2%), compared with the 2011-2014 period (χ^2^ test, *P* < 0.001, Table 4).

**Table 4:** Cardiopulmonary resuscitation survival rates (%) between 2011 and 2017*

Year	Non-survivor	Survivor	Total
2011	143 (95.3)	7 (4.7)	150
2012	162 (90.5)	17 (9.5)	179
2013	144 (86.7)	22 (13.3)	166
2014	187 (84.2)	35 (15.8)	222
2015	201 (74.2)	70 (25.8)	271
2016	193 (77.5)	56 (22.5)	249
2017	164 (80.8)	39 (19.2)	203
Total	1194 (82.9)	246 (17.1)	1440

*χ^2^ test, *P* < 0.001 for the whole cohort; 2011-2013 *P* = 0.008, 2011-2014 *P* < 0.001, 2011-2015 *P* < 0.001, 2011-2016 *P* < 0.001, 2011-2017 *P* < 0.001, 2012-2015 *P* < 0.001, 2012-2016 *P* < 0.001, 2012-2018 *P* = 0.008, 2013-2015 *P* = 0.002, 2013-2016 *P* = 0.018, 2014-2015 *P* = 0.008, other between-year differences not significant.

Multivariate binomial logistic regression assessed the effect of sex, age, initial CPR provider, time for EMS arrival, airway management method, and tourist season on survival rates (represented as OR of survival vs non-survival). Age (OR 1.018, *P* < 0.001), EMS-witnessed CPR (OR vs no BLS performed 3.079, *P* < 0.001, OR vs bystander CPR 2.541, *P* < 0.001), and time for arrival shorter than median time (which was 8 minutes) (OR 1.406, *P* = 0.037) had a significant effect on increased survival rates. Sex, airway management method, and tourist season had no significant effect on survival rates (Table 5).

**Table 5:** Multivariate analysis of factors determining survival – odds ratio of death vs survival at hospital admission. Binomial logistic regression

Factor	Odds ratio (95% confidence interval)	*P*
Male sex	1.34 (0.99-1.82)	0.062
Age	1.02 (1.01-1.03)	<0.001
Initial CPR provider (compared to EMS)		
No BLS performed	3.07 (2.190-4.50)	<0.001
Bystander CPR	2.54 (1.69-3.83)	<0.001
Not documented	1.97 (0.23-17.24)	0.539
Time to arrival >8 minutes	1.40 (1.02-1.93)	0.038
Airway management (compared to ET)		
Face mask ventilation	1.17 (0.55-2.48)	0.691
Nasopharyngeal airway	0.27 (0.04-1.70)	0.162
Oropharyngeal airway	1.39 (0.63-3.07)	0.420
Laryngeal mask airway	0.96 (0.65-1.40)	0.821
Not documented	1.6 (0.90-2.85)	0.112
Tourist season	0.96 (0.71-1.28)	0.761

*CPR – cardiopulmonary resuscitation; BLS – basic life support; EMS – emergency medical service; ET– endotracheal tube.

## Discussion

This study found a gradual improvement in OHCA survival rates in Istra county throughout the studied period (2011-2017), as well as an effect of EMS arrival time, patient age, and initial CPR provider training level on patient survival. Although time for EMS arrival was prolonged during tourist season, there was no significant effect of tourist season on survival rates.

According to the World Health Organization, the quality of cardiac arrest and trauma care provision is a good indicator of prehospital and emergency care (9,10). Here, we assessed the effectiveness of the Institute of Emergency Medicine of Istra County, which makes this study one of the first out-of-hospital resuscitation outcome studies in Croatia. Furthermore, this seven-year follow-up investigation presents a large body of information, reporting survival data after cardiopulmonary arrest for almost 1500 patients.

Survival from OHCA depends on several important factors occurring during different phases of care, including bystander-witnessed arrest, EMS-witnessed arrest, bystander-provided CPR, initial shockable cardiac rhythm, age (<75 years), and return of spontaneous circulation (ROSC) in the field (11,12).

ROSC across European countries ranges from 10% to 50%, and such wide range can be explained by differences in CPR practices and EMS structures (7). Our findings (17.1% survival) fit into this range. What is encouraging is that the number of performed reanimations and their success rate increased throughout the studied period. Of course, this survival rate needs to be perceived in the light of the age structure of the Croatian population, which is among the 10 oldest in the world (13). Therefore, it is reasonable to claim that patients who suffered OHCA had underlying diseases that could have significantly impaired the chances of a successful resuscitation. This is supported by the fact that survivors were significantly younger than non-survivors, which is in accordance with other studies (14-16). Almost 70% of our patients were male, an unsurprising result given that approximately 60% of cardiac arrests occur in men. However, it is not yet clear how gender affects cardiac arrest survival (17), and we may speculate that significantly higher survival rate in women might be a result of protective effects of estrogen and progesterone (18) or earlier symptom recognition.

Further, the mortality rate of OHCA victims increases by 7% to 10% for every minute of delayed BLS (19,20). This emphasizes the importance of prehospital system factors for OHCA survival, particularly time for EMS arrival and bystander CPR initiation, which are the focus of our investigation. The response time interval, defined as the interval from call receipt to patient care, is considered to be the most important component of an efficient EMS system. These intervals vary considerably among EMS systems because of differences in population density, traffic, geography, and EMS units distribution (17). There is a strong correlation between shorter EMS response times and favorable outcomes of cardiac arrest (21). Studies indicate that if ROSC is not achieved within 10 minutes of collapse, BLS efforts will have little to no effect (22). This is why, the International Guidelines 2000 Conference on Cardiopulmonary Resuscitation and Emergency Cardiovascular Care recommend for EMS response time to be under 10 minutes from collapse (23). Although median time from ambulance dispatch to vehicle arrival in this study was in accordance with these recommendations (8 minutes), in the group of OHCA survivors the EMS arrived faster. Also, the survival rates were higher in patients whose first monitored rhythm upon the EMS arrival was shockable (VF or pulseless VT). These rhythms are first monitored in approximately 20% of all cardiac arrests (24).

The standardized management of adult patients in cardiac arrest is described in the ALS guidelines (4,5). The most important intervention affecting survival in patients with shockable rhythms is defibrillation (25-27), preferably less than 5 minutes after arrhythmia onset (23).

Bystanders play an important role as they can provide immediate CPR before the EMS providers’ arrival, which is especially important in remote areas where response intervals are prolonged. Since the absence of bystander-provided initial treatment is associated with extremely low OHCA survival rates (17), the large number of our study participants who did not receive any bystander treatment stresses the importance of general public education in providing bystander CPR. Moreover, we found that BLS education is the key factor affecting CPR outcome, since the proportion of survivors was significantly higher when initial BLS was performed by medical professionals or BLS-educated bystanders. This is an important finding, as it highlights the importance of continuous community-directed BLS education.

Our finding of longer time for EMS arrival during the tourist season comes as no surprise. Although the resuscitation survival rates during tourist season did not differ from those outside of tourist season, for which the EMS staff on duty may be commended, there is an obvious discrepancy between population increase and the number of EMS teams. In order to avoid further increase in onset-to-treatment time and improve outcomes among emergency patients, we suggest continuation of emergency staff education and strengthening community preparedness by organizing CPR training programs, increasing the availability of public-access defibrillators, and allocating additional resources during tourist season.

The study limitations include the fact that the study was conducted in a transitional period for the whole system, which affected medical documentation recording. Utstein registry was introduced in 2013, so all the medical records from the preceding period had to be individually checked, and estimated etiology of cardiac arrest was recorded differently over the years depending on changes in recommendations. The Institutes of Emergency Medicine and Croatian hospitals do not exchange patient data, which is why we did not have insight into the 30-day survival and the patient status in the subsequent treatment. Due to a large employee turnover rate, we did not obtain the number of employees who completed the ALS course, which prevented us from analyzing the effect of education on survival rate. The number of teams on duty was also not recorded in earlier years. However, it must be noted that central EMS dispatcher usually redirects EMS teams from one area to another when the need arises, so the number of teams can be considered to be inversely proportional to the time for EMS team arrival.

The greater survival rate in younger individuals might have been affected by the lower number of comorbidities in this group. Unfortunately, since patient history is not usually fully recorded (one of further factors that needs improvement in pre-hospital emergency system), these comorbidities were not included in the multivariate survival analysis. Comorbidities in survivors and non-survivors have to be retrospectively analyzed to provide a better understanding of factors influencing the CPR outcome.

Although the survival rate after OHCA in Istra County increased, additional strategies might strengthen the “chain of survival.” Education and EMS teams’ training programs should be continuously performed, and young physicians (for many of which EMS is the first workplace after finishing internship) should be encouraged to pursue a career in emergency medicine by being offered professional specialist enhancement. Furthermore, greater emphasis should be placed on BLS education, availability and use of automatic external defibrillators in the community, introduction of state-of-the-art modalities for communication with bystanders, introduction of more EMS teams during tourist season, as well as better communication and medical data exchange with hospitals to obtain feedback about patient outcome.

In recent years Croatia has been facing an exodus of young physicians to western EU countries. The ensuing severe medical staff shortage could be tackled by structured education and training for paramedics, who could perform out-of-hospital interventions and patient stabilization before hospital admission. Also, a nationwide strategy aimed at improving EMS response times and CPR outcome is required. This strategy would need data from studies similar to ours performed in other Croatian counties to inform its development.
